# Ecological Implications of Extreme Events: Footprints of the 2010 Earthquake along the Chilean Coast

**DOI:** 10.1371/journal.pone.0035348

**Published:** 2012-05-02

**Authors:** Eduardo Jaramillo, Jenifer E. Dugan, David M. Hubbard, Daniel Melnick, Mario Manzano, Cristian Duarte, Cesar Campos, Roland Sanchez

**Affiliations:** 1 Instituto de Ciencias Ambientales y Evolutivas, Universidad Austral de Chile, Valdivia, Chile; 2 Marine Science Institute, University of California Santa Barbara, Santa Barbara, California, United States of America; 3 Institut für Geowissenschaften, Universität Potsdam, Potsdam, Germany; 4 Instituto de Ciencias Marinas y Limnológicas, Universidad Austral de Chile, Valdivia, Chile; Argonne National Laboratory, United States of America

## Abstract

Deciphering ecological effects of major catastrophic events such as earthquakes, tsunamis, volcanic eruptions, storms and fires, requires rapid interdisciplinary efforts often hampered by a lack of pre-event data. Using results of intertidal surveys conducted shortly before and immediately after Chile's 2010 *M*_w_ 8.8 earthquake along the entire rupture zone (*ca.* 34–38°S), we provide the first quantification of earthquake and tsunami effects on sandy beach ecosystems. Our study incorporated anthropogenic coastal development as a key design factor. Ecological responses of beach ecosystems were strongly affected by the magnitude of land-level change. Subsidence along the northern rupture segment combined with tsunami-associated disturbance and drowned beaches. In contrast, along the co-seismically uplifted southern rupture, beaches widened and flattened increasing habitat availability. Post-event changes in abundance and distribution of mobile intertidal invertebrates were not uniform, varying with land-level change, tsunami height and coastal development. On beaches where subsidence occurred, intertidal zones and their associated species disappeared. On some beaches, uplift of rocky sub-tidal substrate eliminated low intertidal sand beach habitat for ecologically important species. On others, unexpected interactions of uplift with man-made coastal armouring included restoration of upper and mid-intertidal habitat seaward of armouring followed by rapid colonization of mobile crustaceans typical of these zones formerly excluded by constraints imposed by the armouring structures. Responses of coastal ecosystems to major earthquakes appear to vary strongly with land-level change, the mobility of the biota and shore type. Our results show that interactions of extreme events with human-altered shorelines can produce surprising ecological outcomes, and suggest these complex responses to landscape alteration can leave lasting footprints in coastal ecosystems.

## Introduction

**Figure 1:**
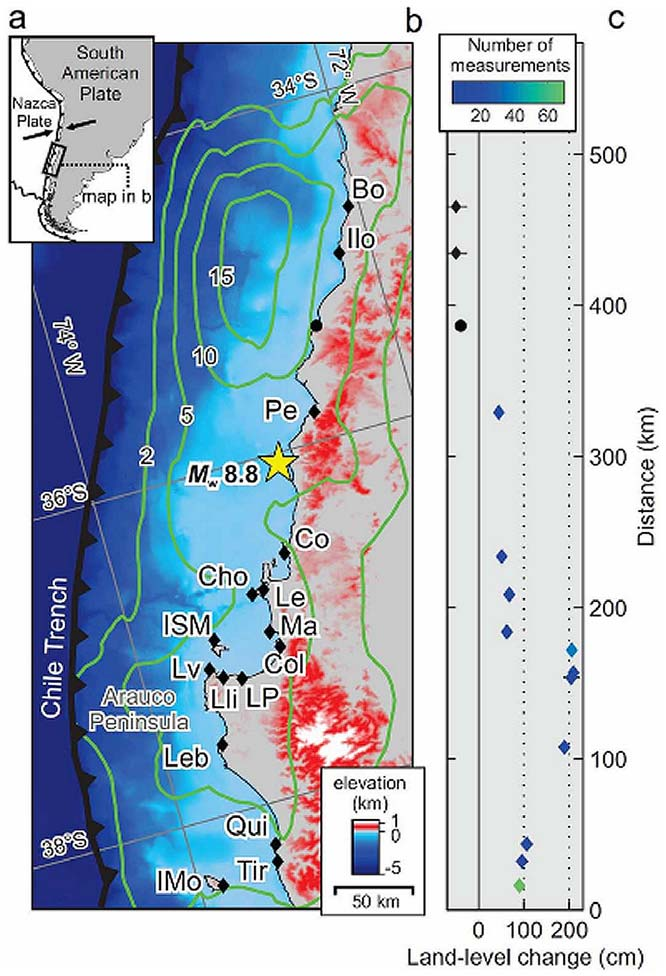
Index maps and land-level changes from the 2010 Maule earthquake. a, Plate-tectonic setting of Chilean margin. Arrows indicate convergences between Nazca and South American plates at *circa* 70 mm yr^−1^. b, Maule earthquake setting. Epicentre indicated by star, green contours show plate boundary slip (m) (10). Beach sites: Boyeruca* (Bo), Iloca* (Ilo), Pelluhue* (Pe), Lenga (Le), Colcura (Col), Punta Lavapie* (Lv), Llico* (Lli), Las Peñas (LP) and Lebu* (Leb). Land-level change estimated at sites with * plus Cocholhue (Co), Chome (Cho), Maule (Ma), Isla Santa María (ISM), Quidico (Qui), Tirúa (Tir) and Isla Mocha (IMo) (Table S2). c, Geographic variability in estimated land-level changes (means ±1 standard deviation). Black dot denotes coseismic subsidence at permanent GPS site CONT [51]; black diamonds are estimates of subsidence based on drowned coastlines.

Chile overlies the convergent boundary between the Nazca plate and the South American continent (Fig. 1a), one of the most seismically active areas on Earth. Along the Chilean margin, plate convergence at ∼70 mm/yr [1] results in great earthquakes (*M_w_*∼8.0–8.5) on average every ∼100–150 years, with occasional giant events (*M_w_*>9.0) every ∼300 years [2], like the 1960 earthquake that reached a moment magnitude (*M_w_*) of 9.5. Such earthquakes result in substantial land-level changes of coastal and inland regions [3], [4], [5], [6]. The plate boundary off central Chile (*ca.* 34–38°S) ruptured last in 1835 and then on February 27, 2010. The 1835 event reached *M_w_*∼8.5 [7], and its effects were documented by Fitz Roy and Darwin, who measured coseismic uplift of 2.4–3.0 m at Isla Santa Maria, southwest of Concepcion [8]. The recent 2010 rupture, known as the Maule earthquake, reached *M_w_* 8.8 becoming the sixth largest event recorded by modern seismology [9], and third in the era of space geodesy following Sumatra 2004 and Japan 2011 [9]. Plate-boundary slip associated with the Maule earthquake reached 20 m, localized mostly in two patches to the north and south of the epicentre [10], [11], [12] (Fig. 1b). The response of the coastline to co-seismic slip depends on its position with respect to these areas of high slip release; thus, subsidence of ∼1 m occurred along the coastline landward of the northern portion (34–35°S), whereas the coast above the southern portion, particularly the Arauco Peninsula (37°S), was uplifted up to 2.5 m [4], [5], [6]. The Maule event triggered a devastating tsunami that killed nearly 500 people [13], destroyed coastal infrastructure, and further reshaped coastal landscapes [4], [5], [6].

Ecological footprints of large-scale extreme events, such as earthquakes, tsunamis, volcanic eruptions, fires and climatic phenomena, can persist for years, decades, centuries and even millennia [14], [15], [16], [17], [18], [19], [20]. For example, the gradation of muddy sedimentary and peaty layers representing coastal land types, suggest that subsidence related to large earthquakes occurred during the Holocene along the outer coast of Washington State [15]. For the same coastal area, analyses of sand deposited over wetland peats point to a tsunami following a large earthquake which probably occurred between 500 and 1700 years ago [17]. The massive wetland created by land subsidence on the Río Cruces after the 1960 *M_w_* 9.5 Chilean earthquake [21], is now recognised as a Ramsar wetland site [22] for its high biodiversity [23], [24]. Despite the dramatic effects of these events, a lack of pre-event and comparative data from diverse ecosystems limits our understanding of their lasting impacts. Moreover, the difficulty of mounting interdisciplinary field investigations immediately following extreme events often prevents collection of ecological information that could lead to important discoveries [25], [26], [27], [28], [29], [30]. For example, results of research initiated nearly 1.5 years after a large disturbance, the severe 1988 fires in Yellowstone National Park in northwest Wyoming, USA, indicating that vegetation in the park was quite resilient surprised ecologists [20], [27]. Similarly, early post-disturbance research following the 1980 Mount St Helen's volcanic eruption, revealed another ecological surprise: vegetation recovery was initiated by propagules which originated within the disturbed areas, rather than outside sources [25].

We provide the first quantification of ecologically relevant effects of a large earthquake and its tsunami, on mobile intertidal invertebrates of sandy beaches by taking advantage of unique results obtained from ecological surveys conducted weeks before and after, as well as over the 10 months following, the Maule earthquake. By incorporating measurements of uplift, subsidence and estimated tsunami heights in our analyses, we provide a broader and more mechanistic perspective on the implications of extreme events for an important coastal ecosystem.

Modification of shorelines and beaches by the construction of seawalls and rock revetments, has for centuries been a common societal response to threats from shoreline erosion, extreme storms and tsunamis [31], [32]. Coastal armouring covers beach habitat, reflects waves and constrains landward migration of the shoreline, leading to decreases in beach width and intertidal habitat [33]. However, despite widespread use of armouring, its ecological impacts to sandy beach ecosystems are only beginning to be considered. Field studies have supported a recent hypothesis suggesting that as beach width narrows in front of armouring structures, habitat is lost disproportionately from the upper and mid-intertidal resulting in loss of biodiversity and ecosystem function [34]. The location of seawalls or revetments on the beach profile and the degree of interaction with waves and tides, are important considerations in predicting impacts; *i.e.* the lower the structure is in the intertidal, the greater both the physical [35] and ecological effects. Importantly, the ecological implications of interactions between coastal armouring and extreme events have never been studied for sandy beaches. For this reason, anthropogenic coastal armouring was included as a key design factor in our study.

Although sandy beaches represent nearly 80% of the ice-free coasts of the world [36], ecological studies (including research on the effects of major disturbances) of this valuable coastal ecosystem have been widely neglected [37]. Thus, our study represents an outstanding opportunity to better understand responses of this economically, culturally and ecologically important coastal ecosystem to extreme events.

## Methods

### Ethics statement

No specific permits were required for the described intertidal field studies. The sandy beaches we studied in Chile are unrestricted to public access and use, and are not privately owned or designated as protected areas (reserves or parks). No protected or endangered species were involved in this study.

In a study designed to evaluate the responses of intertidal beach fauna to armouring we surveyed intertidal zones and animals at nine sandy beaches along the coasts of Maule and Bíobío (*ca.* 34–38°S, Fig. 1b) during late January 2010. We compared biological and physical characteristics of unarmoured sites to those located in front of adjacent seawalls and rocky revetments (Table S1). To quantitatively sample invertebrate macrofauna (defined as animals retained on a 1 mm sieve [38]) we set up four replicated shore-normal transects 5 m apart from each other, extending from the upper intertidal to the low tide level of sites located in front of seawalls or rocky revetments and in unarmoured areas at each beach (see Table S1). Along these transects we sampled each of the three intertidal faunal zones typical of sandy beaches of south central Chile, which are dominated by crustaceans [39], [40]: i) the upper zone occupied primarily by talitrid amphipods (*Orchestoidea tuberculata*), usually extending from the toe of the dunes or natural cliffs to the drift line or high tide level, ii) the mid zone occupied by cirolanid isopods (*Excirolana braziliensis* and *Excirolana hirsuticauda*), extending from the drift line to the effluent line, and iii) the lower zone occupied primarily by hippid crabs (*Emerita analoga*), extending from the effluent line to the lowest tide level or bore collapse line of incoming waves. In each of the three zones present on each replicate transect, five core samples of sediments were collected with a metal cylinder (10 cm in diameter) to a depth of 30 cm at equally spaced levels across the zone, for a total sampling area of 0.04 m^2^ surface area per zone on each transect. The five core samples from each zone in a transect were pooled and sieved through a 1000 µm sieve; afterwards, the collected organisms were stored in 10% formalin in sea water until laboratory sorting. Beach width was measured as the distance between the landward boundary of the beach defined by the toe of foredune or armouring structures and the low tide level during spring tides at each of the four transects at each type of site (armoured, unarmoured). The beach face slope was measured at each of the four transects of each site using the method of Emery [41]. In this study, beach face slope is expressed as 1/x, where x was the distance in meters at which a height difference of 1 m between two consecutive intertidal levels is reached. Thus, higher values of 1/x correspond to flatter beaches.

Following the Maule earthquake and tsunami in February 2010, intertidal surveys as described above were repeated at the nine study sites during March 2010 and used to evaluate responses to the earthquake and tsunami. Comparisons of the abundance of macrofaunal invertebrates between our pre- and post-earthquake and tsunami surveys were made using one way ANOVA on data that were log (n+1) transformed to meet basic assumptions. Changes in beach widths and beach face slopes of sites located in front of seawalls and revetments and in natural unarmoured areas, were calculated as the ratio between the values collected after and before the Maule earthquake (March 2010 and January 2010, respectively). The latitudinal scope of the event and its widespread effects on the coastline, made it impossible to find locations suitable for use as reference sites unaffected by the event. Three beaches from the uplifted zone (Lebu, Llico and Punta Lavapie) were selected for additional surveys during 2010 (July, September and December) based on the intensities of interaction of existing armouring with waves and tides; *i.e.* armouring structures which are located low enough on the beach profile to be reached by waves during high tides experience higher interaction intensity than those located higher on the beach profile.

We measured and compared the magnitude of land-level changes along the study coast (Fig. 1b and Table S2). The magnitude of post-earthquake uplift was determined by measuring the difference in height between the upper growth limit of living rocky shore sessile calcareous algae of the genus *Lithothamnion* (pink coloured) to that of dead organisms (white coloured) which were uplifted above mean sea level [4], [5], [6]. Coastal subsidence estimates were based on measurements of the depths of flooded vegetation in nearby estuaries. We also estimated tsunami height by measuring water marks left on walls and trees; those measurements were corrected for tide level at the time of tsunami arrival.

## Results

Coastal uplift showed a strong geographic pattern, with highest values on the Arauco Peninsula (Punta Lavapie, Llico and Lebu) and Isla Santa María (mean ranges = 189–209 cm). Coastal uplift decreased southward (down to mean values of 96 and 90 cm in Tirúa and Isla Mocha, respectively) and northward (down to 44 cm in Pelluhue). Coastal subsidence (*ca.* −50 cm) affected the two northernmost study sites (Boyeruca and Iloca) (Fig. 1c) drowning the beaches.

**Figure 2:**
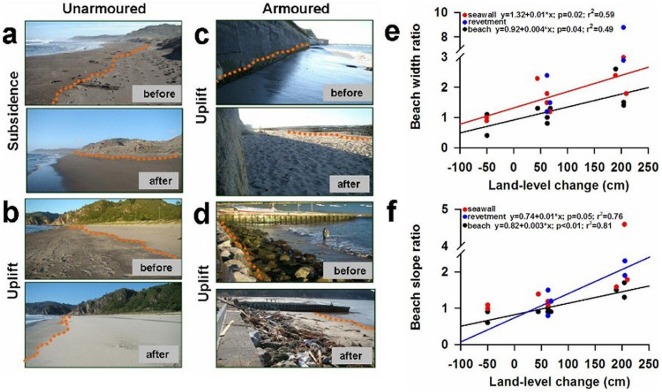
Beach characteristics as a function of land-level changes. a–d, Photos of study sites taken before and after the 2010 Maule earthquake. Orange dotted lines indicate 24 hour spring high tide line; a–b, unarmoured sites: a, subsidence (narrower): Boyeruca, b, coastal uplift (wider): Lebu, c–d, armoured sites: c, seawall with uplifted wider intertidal: Punta Lavapie, d, revetment with uplifted wider intertidal: Llico. Note that dry sand areas above high tide line decreased at subsided beaches (a) and increased at uplifted beaches (b, c and d), e–f, Relationships between the magnitude of land-level changes and e, beach widths and f, beach face slopes for sites with seawalls (red dots), sites with rocky revetments (blue dots) and unarmoured sites (black dots). Only statistically significant regressions are shown.

**Figure 3:**
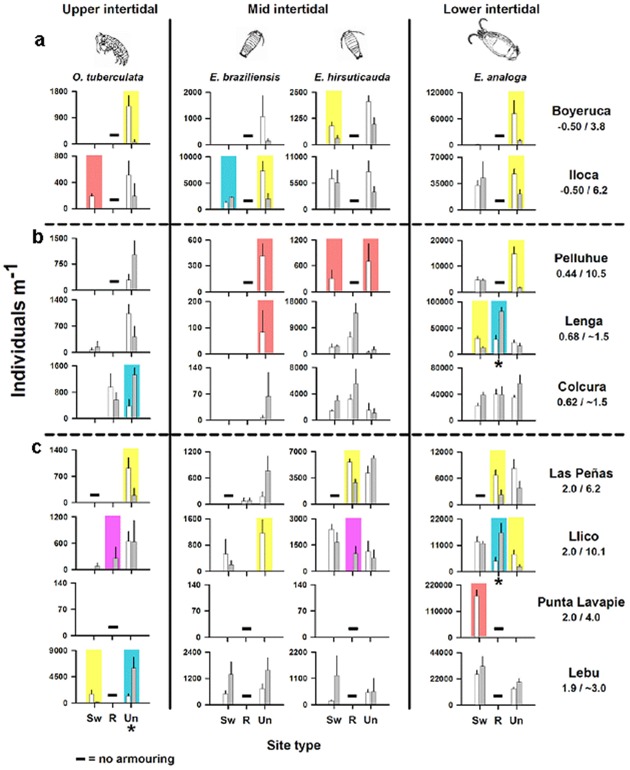
Responses of sandy beach macrofauna to the 2010 Maule earthquake. a, subsided beaches, b, beaches with <1.0 m uplift, c, beaches with 2 m uplift. Mean abundance (+1 standard error) of mobile crustaceans representing upper, mid and lower intertidal zones for before (white bars) and after (grey bars) the Maule earthquake at sites located seaward of seawalls (Sw) and rocky revetments (R), and at unarmoured sites (Un). Shaded rectangles over bars indicate: significant decrease (yellow), disappearance (red), significant increase (light blue) and first appearance of species at a site (purple). The three intertidal zones are represented by 1) the talitrid amphipod *Orchestoidea tuberculata* (upper), 2) the cirolanid isopods *Excirolana braziliensis and Excirolana hirsuticauda* (mid) and 3) the sand crab *Emerita analoga* (lower) as illustrated on the figure. Abundances are presented as individuals m^−1^ to accommodate beach width changes; trends for individuals m^−2^ were similar except three cases indicated by *. Note: low intertidal seaward of the seawall at Boyeruca was bedrock before and after the earthquake, excluding *Emerita analoga*, and the beach at Punta Lavapie is completely armoured with a seawall. Numbers below the names of sites on the right side of the figure indicate land-level changes and tsunami heights in meters (m/m, respectively) for each study beach.

The height of the tsunami ranged from ∼1.5 m to >10 m across the study beaches. Post-earthquake and tsunami changes in beach width and beach slope ratios were significantly correlated with coseismic land-level change for armoured and unarmoured sites (Fig. 2 e, f). In uplifted areas, beaches became wider and flatter by March 2010 and remained so throughout 2010 (Fig. 2 b, c, d). After the earthquake and tsunami we observed significant changes in population abundances of representative taxa of mobile crustacean macrofauna in all intertidal zones, including new appearances, increases, declines and some local extinctions (Fig. 3). These ecological responses varied with land-level change, tsunami height and coastal development. On beaches that subsided and were not armoured, the abundance of all taxa declined in all intertidal zones after the earthquake (Fig. 3a). On armoured beaches with seawalls that subsided, the responses differed among taxa: an upper intertidal beach species (*Orchestoidea tuberculata*) disappeared at Iloca and a lower intertidal beach species (*Emerita analoga*) increased in abundance at the same beach while responses of mid-intertidal taxa varied after the event (Fig. 3a).

The response and direction of change in distribution and abundance of mobile crustacean macrofauna varied with intertidal zone. On uplifted beaches that were not armoured (n = 6) (Fig. 3b, c), declines observed in abundance after the event were more prevalent in the lower intertidal while increases in abundance were more prevalent in the upper intertidal; *i.e.* mean abundance of upper shore taxa increased more than two fold at 50% of sites (Pelluhue, Colcura and Lebu; p<0.05 for the last two sites), while that of lower shore taxa declined by half at 50% of sites (Pelluhue, Las Peñas and Llico; <0.05 for Pelluhue and Llico). In the mid-intertidal, ecological responses were split between increases and declines in abundance, but included disappearance of at least one species (*Excirolana braziliensis*) at two of the sites (Pellhue and Lenga). On uplifted beaches with armouring, increases or no changes in abundance were prevalent compared to declines for upper, mid and lower intertidal zone taxa after the event, especially for sites with revetments (Fig. 3b,c). However, uplifted sites with bedrock in the shallow subtidal, like Punta Lavapie, lost sandy intertidal zones and macroinvertebrate fauna (*Emerita analoga*) due to uplift of rocky substrate into the low intertidal. This is somewhat analogous to ecological effects of armouring on upper and mid-intertidal zones (34); *i.e.* the uplifted bedrock replaced the swash zone habitat, and the lower intertidal sand crab *Emerita analoga* disappeared. Ecological effects of the tsunami, such as those from washover and scour, were less clear; however, the lower intertidal sand crab, *Emerita analoga* declined significantly (p<0.05) on both unarmoured sites where tsunami heights were ≥10 m (Pelluhue and Llico; Fig. 3 b,c). In addition, the tsunami height was 10.5 m at the only site (Pelluhue) where disappearance of both mid-intertidal taxa (*Excirolana braziliensis* and *Excirolana hirsuticauda*) was observed (Fig. 3b).

**Figure 4:**
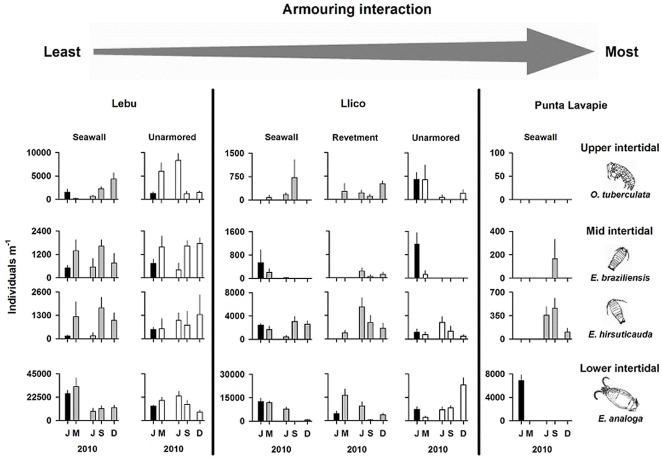
Responses of sandy beach macrofauna to uplift and coastal armouring over time. Temporal patterns in population abundance of crustaceans from the three intertidal zones listed in Fig. 3 for armoured sites that experienced different amounts of interaction between the structure and local waves and tides before the 2010 earthquake and tsunami, and for unarmoured sites. Mean values (+1 standard error) of abundance before the event are indicated by black bars, mean values of post-event abundance for armoured sites are grey bars and unarmoured sites are white bars. Strength of pre-event armouring and wave interaction (see Methods) increases from left to right as indicated by the grey arrow at the top of the figure. Data are presented as individuals m^−1^ to accommodate changes in beach widths; trends for individuals m^−2^ were similar.

**Figure 5:**
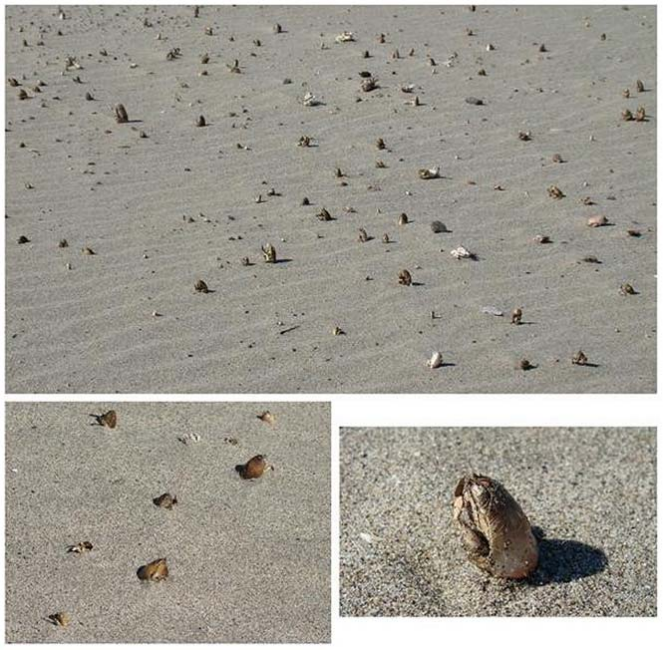
Dead sand crabs, *Emerita analoga*, stranded in life position at the upper-shore levels of the armoured site at Lebu, illustrate the direct mortality of a dominant lower intertidal invertebrate species observed immediately after the Maule earthquake and tsunami.

Results of continuing surveys of uplifted beaches during 2010 indicated different levels of recolonization, estimated by variability in species composition and population abundance of macroinvertebrates of the intertidal zones, on sites located in front of armouring. These patterns were related to the intensity of interaction of the armouring structures with waves and tides before the earthquake. In front of a seawall that did not interact with waves and tides (Lebu), post-earthquake species composition was the same as pre-event (Fig. 4). For all except one species (*Emerita analoga*), population abundances were similar or even higher than before February 27^th^ and remained similar for 10 months following the earthquake and tsunami at Lebu (Fig. 4). Although when the tsunami hit the seawall it appeared to cause high mortality of the larger size classes of the lower intertidal crab *Emerita analoga* (Fig. 5) (the most abundant species prior to the earthquake), by the time of our post-event survey in March, the overall abundance of this species was higher than before the earthquake. However, in all subsequent months the abundance of *Emerita analoga* was nearly 50% lower than before the earthquake and tsunami at that site (Fig. 4). At the unarmoured site on this beach, although post-earthquake species composition remained the same as before the earthquake for the rest of 2010, abundance varied considerably in two of the four taxa we studied (*Orchestoidea tuberculata* and *Excirolana braziliensis*; Fig. 4). In front of a formerly interacting seawall (Llico), species composition was the same before and immediately after the earthquake (Fig. 4). However, population abundance of *Excirolana braziliensis* was lower immediately following the earthquake (March), and later this species disappeared (Fig. 4). At the same site, population abundance of *Orchestoidea tuberculata* was higher in surveys five and seven months after the earthquake (July and September) but disappeared during December 2011 (Fig. 4) after the beach was cleared of tsunami debris by heavy equipment. In front of a revetment at Llico, only one species, the lower intertidal *Emerita analoga*, was present before the earthquake (Fig. 4). One month after the earthquake, the intertidal habitat restored in front of this revetment by uplift was colonized by upper and mid-intertidal species (*Orchestoidea tuberculata* and *Excirolana hirsuticauda*), and then *Excirolana braziliensis* by July (Fig. 4). Thus, five months after the earthquake this armoured site had regained the typical species composition of unarmoured sandy beaches of south central Chile (38,39). In front of the armouring with the highest interaction with waves, the seawall at Punta Lavapie, again only one species, the lower intertidal *Emerita analoga* was present before the earthquake (Fig. 4). Five to seven months after (July and September), mid-intertidal isopods (*Excirolana braziliensis* and *Excirolana hirsuticauda*) appeared for the first time on the restored intertidal habitat at this site. In contrast, formerly abundant lower intertidal sand crabs *Emerita analoga* disappeared right after 27 February (Fig. 3) coincident with the uplift of subtidal rocky substrate; very low numbers were observed during September and this species disappeared again in December 2010 (Fig. 4).

## Discussion

An upward trend in the frequency of major natural disturbances across different ecosystems around the world has been suggested by a number of models and sources [42], [43], [44], [45]. In fact, the annual number of major natural disturbances that have significantly affected society increased four fold from 1975 to 2008 [29], [46]. Major earthquakes (recently in Haiti, Chile and Japan) causing tragic loss, economic devastation and destruction during the last few years, emphasize the high vulnerability of the world's heavily populated and ecologically critical coasts. Pressing needs for societal recovery can overshadow the urgency of understanding complex effects of large-scale disasters on coastal ecosystems. In this study, we took advantage of the unique opportunity to document the complex and surprising ecological responses of an important and widespread coastal ecosystem, sandy beaches, to a major earthquake and tsunami for the first time. Our novel results indicate that the extreme earthquake and tsunami of 27^th^ February 2010, resulted in significant and lasting changes in physical and ecological attributes of sandy beaches on the affected coast. The responses of ecologically important species that are characteristic of the upper, mid and lower intertidal zones of beaches in the region suggest the entire intertidal zone was affected on some beaches. The changes we observed in the distribution and abundance of mobile intertidal crustaceans after the Maule event (including new appearances, increases, declines and some local extinctions), were much greater than would be expected during mid-summer when sandy beaches are generally most stable and in the season of sediment accretion. Our findings also illustrate how current management of coastal urbanized zones can result in significant deterioration of sandy beach ecosystems [33], [34], [38], [47].

The most unexpected changes observed in sandy beach invertebrates after the earthquake and tsunami, occurred in front of artificial shore armouring along the uplifted coast of the Arauco Peninsula. Where coastal armouring structures constrained landward extent of the sandy beach, increases in intertidal width created new habitat that was rapidly colonized by upper-intertidal and mid-intertidal species via migration from adjacent unarmoured beach areas. Thus, restoration of habitat in front of seawalls and rocky revetments by uplift had positive effects on mobile upper-intertidal and mid-shore macrofauna: *Orchestoidea tuberculata* and *Excirolana hirsuticauda* appeared for the first time in front of the revetment at Llico right after the Maule earthquake while *Excirolana braziliensis* also recolonized the area, but after five months (Fig. 4). Similarly, both species of *Excirolana* colonized the uplifted intertidal zone in front of the seawall at Punta Lavapie, a zone formerly occupied by just the lower intertidal crab *Emerita analoga* (Fig. 4). Nearly eight months after the Maule earthquake (October 2010), native and exotic coastal strand vegetation began to colonize the new supralittoral zones formed by uplift on sandy beaches with armouring structures (Jaramillo, unpublished). The appearance of perennial vegetation that can potentially trap wind-blown sand to form hummocks and embryonic dunes, could further transform these coseismically uplifted coastal habitats to provide an important ecotone between intertidal and terrestrial ecosystems that was eliminated by armouring prior to the earthquake. Such coastal strand zones have become increasingly rare on developed coastlines and are threatened by sea level rise [47], [48].

Although ecological effects of the tsunami on sandy beach invertebrates were challenging to isolate from the effects of uplift or subsidence, high mortality of intertidal animals were associated with large tsunami heights (>10 m), for example at Pelluhue where mid-intertidal species disappeared (*Excirolana braziliensis* and *Excirolana hirsuticauda*) or decreased significantly in abundance (*Emerita analoga*) after the Maule earthquake. Thus, our results suggest that even in the absence o coseismic uplift or subsidence, the ecological effects of tsunamis on beach ecosystems could be significant as been shown for subtidal soft bottom macrofauna [30]. How lasting those effects may be, will depend on the species assemblage affected and proximity of source populations, as well as shoreline evolution and human responses to the disaster. For example, weeks after the Indian Ocean tsunami of December 24^th^ 2004, which removed large amounts of sediments along sandy beaches and dunes of the coast of northwestern Sumatra, a new coast begun to form with similar features to the pre-event period. However, beach recovery was hindered by coastal development, including the installation of new armouring structures in the form of rocky revetments [49], [50].

As predicted [34], our pre-earthquake surveys found that the upper and mid-intertidal species (*Orchestoidea tuberculata*, *Excirolana braziliensis* and *Excirolana hirsuticauda*) of sandy beaches are more strongly affected by armouring than lower shore species (*Emerita analoga*) and that the degree of interaction with waves and tides influenced these ecological impacts on sandy intertidal animals. After the earthquake, these upper and mid-intertidal mobile species, all of which have direct development and thus low dispersal, quickly expanded distributions and increased in abundance in response to uplift that altered beach profiles and reduced interaction of the armouring structures with waves and tides. We hypothesise that this rapid response was possible due to the proximity of resident source populations living on adjacent unarmoured sections of the study beaches. For coastal areas where armouring is more extensive and for beaches that are isolated from potential source populations, responses of these intertidal species with low dispersal would be expected to be considerably slower. In contrast, the sand crab *Emerita analoga*, a species with free swimming larvae and high potential for dispersal, had not recovered from mortality due to the earthquake and tsunami at some sites after nearly a year due to extreme habitat change, specifically the uplift of rocky substrate which replaced the sandy habitat in the low intertidal zone of Punta Lavapie.

Ecological effects of extreme events, such those we observed for the Maule event, are expected to vary in duration. Shorter-term effects on beaches included direct mortality associated with the tsunami (Fig. 5) and the indirect bottom up effects of increased inputs of algal wrack from uplifted rocky shores on upper shore invertebrate consumers such as talitrid amphipods (*Orchestoidea tuberculata*). However, in areas with significant uplift (*ca.* 2 m), locations of armouring structures were shifted higher on the beach profile, reducing interaction with waves and tides and restoring intertidal zones for biota and ecological function. For this reason we expect positive changes observed in these beach ecosystems to persist, altering intertidal community composition and dynamics over the long term, even in front of existing coastal armouring. In contrast, for the subsided armoured areas, community composition and population abundances are expected to remain depressed over time.

Responses of mobile sandy beach invertebrates to the earthquake and tsunami differed dramatically from that observed on adjacent rocky shores where uplift caused massive mortality of sessile biota, including macrophytes [4], [5], [6]. Thus, ecological impacts of extreme events on coastlines appear to vary strongly with magnitude and direction of land-level change, across shore types and with the mobility of the biota. Our results emphasize the value of and need for baseline information on coastal ecosystems, and illustrate how interactions of extreme events with human-altered coasts can produce unexpected ecological outcomes. Understanding how these complex and habitat-specific responses to episodic extreme landscape alteration can create lasting ecological signatures, provides important new insights on processes and dynamics of coastal ecosystems.

## Supporting Information

Table S1 **Geographic coordinates of the sandy beaches studied indicating the types of sites sampled at each beach.**
(DOC)

Table S2 **Geographic coordinates of the rocky shore sites visited to estimate land-level changes.**
(DOC)
