# Supplementary material for: Ecological Implications of Extreme Events: Footprints of the 2010 Earthquake along the Chilean Coast
Source: PLoS One. 2012 May 2;7(5):e35348. doi: 10.1371/journal.pone.0035348 (PMC3342270; doi:10.1371/journal.pone.0035348)
Supplement: Table S1 — Geographic coordinates of the sandy beaches studied indicating the types of sites sampled at each beach. (DOC) [file pone.0035348.s001.doc]

Table S1. Geographic coordinates of the sandy beaches studied indicating the types of sites sampled at each beach.

|  |  | | sampling sites | | |
| --- | --- | --- | --- | --- | --- |
|  |  |  |  |  |  |
| sandy beaches | south latitude | west longitude | unarmoured  sites | sites in front of seawalls | sites in front of rocky revetments |
|  |  |  |  |  |  |
| Boyeruca | 34°41'32.5" | 72°03'43.8" | X | X |  |
| Iloca | 34º56'26.8'' | 73º11'07.7'' | X | X |  |
| Pelluhue | 35°48'56.5" | 72°35'12.8" | X | X |  |
| Lenga | 36°45'51.6" | 73°10'28.3" | X | X | X |
| Colcura | 36°45'51.6" | 73°10'28.3" | X | X | X |
| Punta Lavapie | 37°08'52.0" | 73°34'54.5" |  | X |  |
| Llico | 37°11'10.8" | 73°33'54.4" | X | X | X |
| Las Peñas | 37º14'25.0'' | 73º25'29.8'' | X |  | X |
| Lebu | 37º35'8.6'' | 73º38'37.8'' | X | X |  |
