# Supplementary material for: Ecological Implications of Extreme Events: Footprints of the 2010 Earthquake along the Chilean Coast
Source: PLoS One. 2012 May 2;7(5):e35348. doi: 10.1371/journal.pone.0035348 (PMC3342270; doi:10.1371/journal.pone.0035348)
Supplement: Table S2 — Geographic coordinates of the rocky shore sites visited to estimate land-level changes. (DOC) [file pone.0035348.s002.doc]

Table S2. Geographic coordinates of the rocky shore sites visited to estimate land-level changes.

| rocky shore sites | south latitude | west longitude |
| --- | --- | --- |
|  |  |  |
| Boyeruca | 34º41’22.2’’ | 72º03’39.1’’ |
| Iloca | 34º56’28.4’’ | 72º11’09.9’’ |
| Pelluhue | 35º48’56.1’’ | 72º35’10.7’’ |
| Cocholhue | 36º35’53.3’’ | 72º58’31.9’’ |
| Chome | 36º46’07.1’’ | 73º12’29.9’’ |
| Maule | 37º00’18.6’’ | 73º11’04.1’’ |
| Isla Santa María | 37º01’33.6’’ | 73º31’01.8’’ |
| Punta Lavapie | 37º09’02.8’’ | 73º34’23.2’’ |
| Llico | 37º11’00.3’’ | 73º33’50.6’’ |
| Lebu | 37º35’24.3’’ | 73º38’48.4’’ |
| Quidico | 38º14’09.1’’ | 73º28’21.0’’ |
| Tirúa | 38º20’10.3’’ | 73º29’52.1’’ |
| Isla Mocha | 38º22’18.8’’ | 73º54’41.7’’ |
